# Malignant transformations in mature cystic teratomas: A case report and literature review

**DOI:** 10.3892/mi.2025.251

**Published:** 2025-07-07

**Authors:** Oğuzcan Özkan, Asli Geçgel, Pinar Peker, Erhan Gökmen

**Affiliations:** 1 Department of Medical Oncology, Ege University Faculty of Medicine, 35100 Izmir, Turkey; 2 Department of Medical Oncology, Adana State Hospital, 01170 Adana, Turkey

**Keywords:** malignant transformation, mucinous adenocarcinoma, somatic-type malignancy, teratoma, testicular cancer

## Abstract

Testicular teratomas, although generally benign, may rarely undergo malignant transformation into somatic cancers, most notably, adenocarcinomas. The incidence of malignancy transformation in ovarian teratomas is well-established at ~2%; however, it is extremely uncommon for testicular and extragonadal teratomas to undergo such transformations. The present study describes a unique case of a testicular teratoma that underwent malignant transformation into mucinous adenocarcinoma. The pathophysiology underlying testicular teratoma transformation remains incompletely understood, with several mechanisms proposed, including the malignancy of totipotent embryonal carcinoma cells or the malignant differentiation of mature teratomatous elements. Additionally, secondary malignancy, often induced by chemotherapy or radiotherapy, is also a contributing factor in certain cases. Immunohistochemical analyses play a crucial role in diagnosing these rare malignancies, with markers such as CDX2, CK20 and CK7 helping to differentiate the adenocarcinoma phenotypes. Despite the rarity of such cases, it is imperative to recognize the potential for aggressive progression, particularly when adenocarcinoma is involved, as these cases require intensive management, including chemotherapy and, sometimes, surgical intervention. The present study emphasizes how crucial early discovery is for improving prognosis and treatment results for individuals with malignant transformation of testicular teratomas, as well as the value of a multidisciplinary approach. Given the limited number of reported cases and lack of standardized management guidelines, the timely documentation and analysis of such rare presentations are essential to guide future clinical decision-making.

## Introduction

Teratomas are tumors of embryonic origin classified within the group of non-seminomatous germ cell tumors (GCTs) (1). While they are most frequently detected in the ovaries or testes, they can also be found in the mediastinum, retroperitoneum and central nervous system (2). Non-seminomatous GCTs can manifest as pure teratomas; however, they are more commonly observed in mixed forms that involve various non-seminomatous histologies. The only histological element found in the residual lesions following platinum-based treatment for metastatic mixed non-seminomatous GCTs are teratomas. Mature cystic teratomas (MCTs), also known as dermoid cysts, are benign GCTs comprised of mature tissues that originate from the endoderm, mesoderm, or ectoderm layers of the embryo. They comprise ~20% of all ovarian tumors and 95% of all ovarian GCTs (2,3). These tumors contain totipotent stem cells that can transform into a wide range of tissue types, including mesodermal tissues such as bone and muscle, endodermal tissues present in the lungs and gastrointestinal tract, and ectodermal tissues such as hair and skin. The most widely accepted theory regarding their origin suggests that teratomas arise from primitive germ cells (2). Teratomas are the most common type of testicular GCTs in children (3). In this age group, mature teratomas usually present with a benign clinical course. However, in adults, teratomas tend to exhibit metastatic potential. Clinically, mature teratomas are generally slow-growing tumors, with their clinical presentations varying significantly. Acute abdominal pain from ovarian teratomas may occur as a result of ovarian torsion or spontaneous rupture, as well as nonspecific abdominal-pelvic discomfort and compressive effects on nearby organs (4,5). The present study aimed to address this gap by presenting a rare case and discussing its implications for pathophysiological understanding and clinical management.

The differentiation into somatic-type tissues that reflect either embryonic or adult developmental stages distinguishes teratomas from other types of neoplasms (6,7). Males who are prepubertal or postpubescent may develop testicular teratomas; however, the biological behavior and prognosis of these tumors vary significantly between these age groups. Teratomas in children are usually diagnosed prior to the age of 4 years, are often pure and are generally benign (8,9). These prepubertal tumors fall under the category of non-GCTs and are not linked to chromosomal abnormalities such as the presence of isochromosome 12p (i12p) or germ cell neoplasia *in situ* (GCNIS). Adult postpubertal teratomas, on the other hand, are typically parts of mixed GCTs. They can be found in metastatic locations, particularly after non-teratomatous GCT components have received chemotherapy. This theory is supported by a high degree of genetic concordance between teratomas and GCT metastases from the same patient (10). Notably, elevated serum levels of tumor markers such as β-human chorionic gonadotropin (β-hCG) or alpha-fetoprotein (AFP) indicate the presence of other co-existing GCT components rather than being caused by the teratomatous elements themselves.

Teratomas have been divided into four subtypes for both descriptive and diagnostic reasons, with an increasing focus on understanding their *pathophysiological basis* in recent literature. Since both types are prone to somatic-type malignant transformation and can metastasize, this distinction has limited clinical significance (10). Organoid structures and differentiated somatic tissues comprise mature teratomas, which are usually embedded in a fibrous or myxoid stroma (6,7). Skin appendages, thyroid follicles, pancreatic tissue, respiratory and gastrointestinal epithelium, cartilage, as well as squamous epithelium are examples of common constituents. Although they are common in the ovary, MCTs are uncommon in the testis. These typically have no immature components and are composed only of sebaceous glands and squamous epithelium. Similarly, there may be epidermoid cysts, which are comprised entirely of keratinized squamous epithelium and do not have adnexal structures. Since teratomas are usually linked to this chromosomal abnormality and have the potential to become malignant, the lack of i12p can aid in differentiating these lesions from malignant teratomas (11). With an average presentation age of 24 years, a small subset of dermoid cysts and teratomas identified after puberty appear benign (9). These tumors are tiny, cystic, unrelated to GCNIS and are frequently found close to the testicular hilum. Additionally, every case examined using fluorescence *in situ* hybridization (FISH) for i12p has yielded negative results (10). In any case, these tumors usually have a benign clinical course. The presence of embryonal or poorly differentiated tissues characterizes immature teratomas. Although primitive neuroepithelial elements are frequently regarded as immature components, the standards for determining the degree of immaturity are still unclear. Although these characteristics are present, their clinical significance remains unclear, and adult pathology reports frequently undervalue them (12). Histological characteristics suggestive of somatic malignancy characterize malignant transformation within teratomas. Large-scale growth of immature neuroectodermal components that resemble embryonal tumors may be one example of this (13). Malignant transformation may be detected with the help of imaging modalities like MRI and ultrasound. While MRI may show fat suppression or enhancement patterns suggestive of neoplastic tissue, ultrasound findings suggestive of malignancy include complex echotexture with branching isoechoic elements (14). Although they lack specificity, serum biomarkers such as carcinoembryonic antigen (CEA) and carbohydrate antigen 19-9 (CA 19-9) may bolster clinical suspicion of malignancy (15). Although it is less informative in other histologic subtypes like intestinal adenocarcinoma, elevated serum SCC antigen (SCC-Ag) may be seen in cases of transformation to squamous cell carcinoma (SCC) and can be useful in the diagnostic process (16,17). Definitive diagnosis requires histopathological analysis, despite suggestive imaging and tumor markers. In the present study, in reviewing the literature, case reports published within the past 20 years were selected based on the availability of histopathological confirmation and relevance to postpubertal testicular teratomas.

Other than observation, localized testicular teratomas usually do not require any treatment. Advanced cases, however, require additional care. There is no standard chemotherapy regimen available as malignant transformation is uncommon, and there is limited reproducibility across reported protocols due to heterogeneity in histological presentation. However, 5-fluorouracil and platinum-based chemotherapy have shown promise in certain instances (13,18). The identification of KRAS mutations in certain patients raises the possibility that targeted EGFR therapies could be an effective course of treatment (19). Tumor extension outside the testis, invasion of the cyst wall, ascites, spontaneous or iatrogenic rupture, adhesion to surrounding tissues and non-SCC malignant histologies are all poor prognostic factors that are linked to aggressive disease and poorer outcomes (20). It is extremely uncommon for testicular GCTs to malignantly transform into adenocarcinomas. The present study describes a rare instance of testicular metastatic teratoma that transformed into intestinal-type mucinous adenocarcinoma, a phenomenon that, to date, at least to the best of our knowledge, has not been reported in the literature.

## Case report

A 52-year-old male patient with no known history of chronic illnesses or malignancies in his family presented to the Urology Clinic at Ege University Hospital (Izmir, Turkey) with swelling in the right testis, which had developed 21 years prior (June, 2004). At that time, the levels of serum tumor markers were elevated with an AFP level of 550 ng/ml, a β-HCG level of 1,826 IU/l and a lactate dehydrogenase (LDH) level of 1,313 U/l. A scrotal Doppler ultrasound revealed a necrotic, vascularized mass in the right testis, measuring ~6 cm in diameter. A whole-body CT scan revealed peripancreatic, paraaortic and aortocaval conglomerate lymphadenopathies (since these CT images were obtained 21 years ago and were obtained at an external center, the actual images could not be retrieved; thus, only the report information has been shared). The patient underwent a right radical orchiectomy, and a histopathological examination (images not available) revealed findings consistent with embryonal carcinoma. The tumor, measuring 6 cm, exhibited widespread lymphovascular invasion and invasion of the tunica albuginea. No evidence of metastasis was found in the lungs or brain at initial staging, although significant retroperitoneal involvement was noted. Due to advanced-stage disease, the patient received four cycles of bleomycin, etoposide and cisplatin (BEP) chemotherapy (typically consisting of bleomycin 30 units/m² once weekly for 3 weeks, etoposide 100 mg/m² daily for 3 consecutive days, and cisplatin 20 mg/m² daily for 5 consecutive days administered in 3-week cycles) between July and October, 2004, during which partial regression of the lymph nodes was observed. During chemotherapy, the patient developed neutropenic fever and required hospitalization for supportive care, including the immediate initiation of granulocyte colony-stimulating factor via subcutaneous injection at a dose of 5 mcg/kg/day, continued daily until the absolute neutrophil count recovered above 1,000/mm³. Following chemotherapy, a residual tumor measuring 2.5 cm was found, prompting a retroperitoneal lymph node dissection (RPLND), which revealed findings consistent with a mature teratoma in November 2004. At 3 months following surgery (February 2005), the patient developed elevated AFP levels, and a 2.5 cm retroperitoneal lymph node was detected, leading to the administration of four cycles of VIP (etoposide, ifosfamide and cisplatin) chemotherapy. The VIP regimen consists of etoposide 75 mg/m² administered intravenously daily on days 1 through 5, ifosfamide 1,200 mg/m² intravenously daily on days 1 through 5 with mesna uroprotection, and cisplatin 20 mg/m² intravenously daily on days 1 through 5, repeated every 3 weeks. Partial regression was again noted, and the patient was subsequently placed under follow-up care. The VIP regimen was complicated by grade 2 neurotoxicity and transient renal function deterioration, both of which resolved with dose adjustment and hydration protocols.

**Figure 1:**
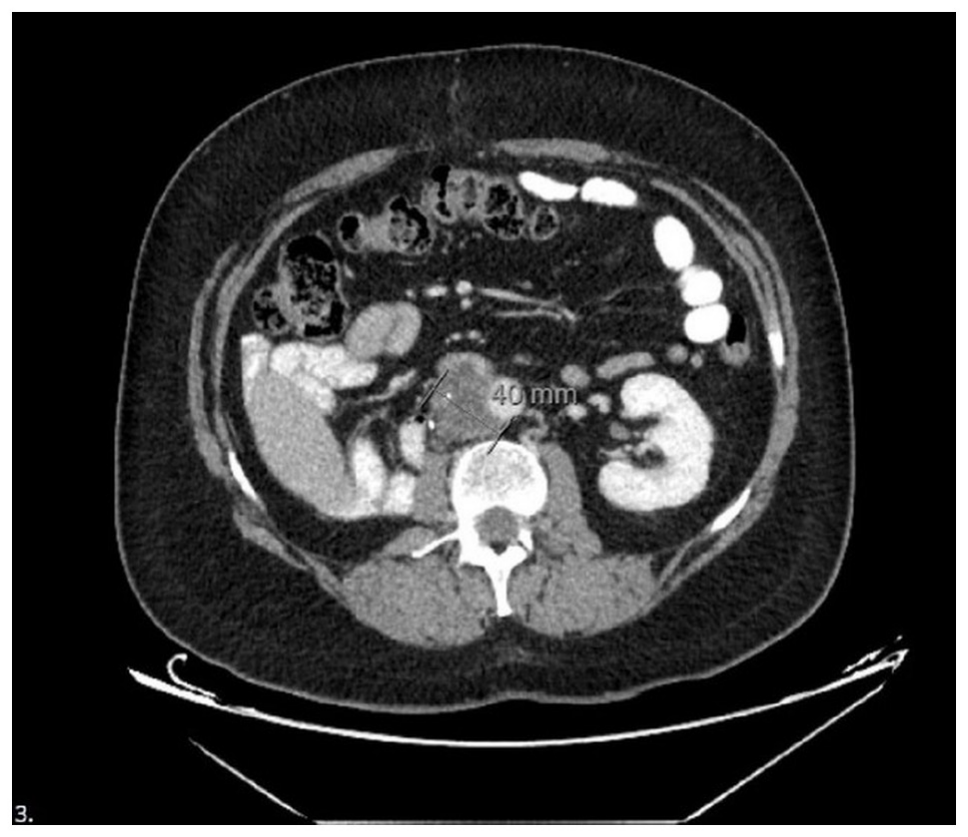
Computed tomography image of the retroperitoneal mucinous adenocarcinoma lesion arising from a teratoma. Contrast-enhanced axial CT image demonstrating a heterogeneous retroperitoneal mass with cystic and solid components, consistent with teratoma-derived adenocarcinoma following chemotherapy for relapsed testicular germ cell tumor. In this CT image, the tumor along with a 40-mm lymphadenopathy (LAP) complex that was developed as a result can be observed.

At 9 years post-operatively (November, 2013), the patient experienced AFP progression, prompting a CT scan of the thorax and abdomen, which again revealed a 2.5-cm retroperitoneal lymph node (it should be noted that only the reports of the CT images taken during this period are available for the patient. The actual images could not be obtained because they were performed at a private radiology laboratory in an external center). RPLND was performed, and the pathological examination was consistent with a mature teratoma. The immunohistochemical findings were as follows: AFP, positive; CD30, negative; PLAP, positive; OCT-4, negative; and D2-40, negative; this confirmed the diagnosis of a mature teratoma. Samples were obtained from different retroperitoneal lymph node regions during RPLND and were separately labeled and processed for histopathological evaluation. In the pathology report, samples 1 and 3 exhibited cystic structures lined with single-layered or cuboidal epithelium. Samples 2 and 4 revealed epithelial cysts and chondroid areas, which were consistent with the teratomatous component of the GCT. Samples 7 and 8 displayed areas of fat necrosis, thrombus organization, giant cells and inflammatory cell infiltration with vascular proliferation, which were interpreted as secondary changes. A reactive lymph node was dissected from sample 5. Sample 4 exhibited solid epithelial tumors along with cystic structures, and immunohistochemical analysis identified these as compatible with the solid yolk sac component of the tumor. All specimens contained only tumor tissue, with no surrounding lymphoid tissue. Of note, the pathological blocks were assessed at a private external laboratory by medical pathologists experienced in uro-oncology. Therefore, detailed visual and pathological data could not be provided. The information we share is based solely on the pathology report. The patient then received an additional four cycles of VIP chemotherapy between December, 2013 and March, 2014. Follow-up imaging performed 8 years later revealed new abdominal findings, including a 56 mm segment of a lobulated soft tissue mass in the pericaval region, reaching a size of 4 cm. The mass caused the obliteration of the vena cava lumen at the caudal level, with no contrast observed within the lumen, indicating venous compression and possible thrombosis. This vascular invasion led to bilateral lower extremity edema and venous stasis symptoms, requiring low-molecular-weight heparin therapy. These findings were compared with prior imaging reports from June, 2018, which revealed no such soft tissue mass. The current images revealed the development of recurrent lymphadenopathy (LAP) metastasis and vena cava invasion (Fig. 1).

The mass, which also infiltrated the abdominal aorta, underwent an incisional biopsy by the cardiovascular surgery team. During this period, the serum levels of tumor markers including AFP (normal range, 0-10 ng/ml), β-HCG (normal range, <5 IU/l) and LDH (normal range, 140-280 U/l) were within normal limits. A pathological examination revealed that immunohistochemical stains for AFP, CD30, glypican, OCT-4 and SAL-4 were all negative. The resected material exhibited a mucinous adenocarcinoma component along with a benign chondroid component. These findings raised the possibility of mucinous adenocarcinoma arising from a teratoma. This represented a rare instance of somatic-type malignant transformation, a known but infrequent complication of mature teratomas. Following the pathological diagnosis, a gastrointestinal tract screening was performed, specifically an endoscopic colon examination, which revealed no abnormal findings. Following radiation therapy targeting the residual mass, the patient received four cycles of adjuvant chemotherapy with an oxaliplatin + capecitabine-based regimen (XELOX) from May to November, 2023. The XELOX regimen consisted of oxaliplatin 130 mg/m² administered intravenously on day 1, and capecitabine 1,000 mg/m² taken orally twice daily on days 1 through 14 of each 3-week cycle. Due to significant neuropathy symptoms, treatment was completed with four cycles of capecitabine over a period of 6 months. Despite cumulative treatment burden, the patient maintained an adequate performance status and responded well to supportive interventions, including neuroprotective agents. In the third postoperative year, the patient remains under surveillance with no evidence of recurrence.

## Discussion

In primary testicular tumors, teratomas rarely undergo somatic malignant transformation. Somatic malignancy transformation refers to the development of a non-GCT within a mature teratoma. While the malignant transformation of ovarian cystic teratomas has been well-documented, with an incidence of ~2%, the occurrence of such transformations in mature teratomas outside the ovaries, including in the testis, is exceedingly rare (21). However, comparisons with prior studies on malignant transformation in testicular teratomas remain limited, highlighting a need for further research in this area. In the existing literature, only a limited number of cases of malignant transformation in primary testicular teratomas have been reported. One of these cases involved colon transformation, while the other two lacked specific pathological details. Notably, the transformation of testicular teratomas into signet ring adenocarcinoma has been described in only a single case (22).

The mechanism underlying the malignant transformation of testicular mature teratomas remains incompletely understood. Sarcoma is the most commonly reported malignancy; however, various other tumor types, including enteric adenocarcinoma, primitive neuroectodermal tumors and leukemia, have also been observed (6,23-25). Furthermore, a previous study reported the transformation of testicular GCT metastasis into papillary renal cell carcinoma following platinum-based chemotherapy (26). Of note, two main mechanisms have been proposed for malignant transformation. One of these mechanisms suggests that malignancy arises from the differentiation of totipotent embryonal carcinoma cells into somatic phenotypes, while the other points to the malignant transformation of mature teratoma components themselves (27). Despite these insights, stronger referencing to recent studies is required to support these proposed mechanisms fully. Another crucial factor in testicular teratoma malignancy transformation is the potential secondary transformation due to chemotherapy or radiotherapy. The literature indicates that only a few cases of testicular retroperitoneal metastatic GCTs undergo malignant transformation due to chemotherapy or radiotherapy, some of which exhibit sarcomatoid changes (23). In addition, there have been reports of malignant transformations in untreated primary testicular teratomas (6,20,23). Notably, the adenocarcinoma phenotype-related malignant transformation in primary mature testicular teratomas remains exceptionally rare.

It is an uncommon occurrence for somatic-type cancers, specifically intestinal-type adenocarcinomas, to develop within testicular teratomas. It is considered that endodermal components that resemble the epithelium of the lower gastrointestinal tract are the source of these cancers. Such tumors often express markers of intestinal differentiation, such as CK20 and CDX2, according to immunohistochemical analyses (24). The identification of these markers lends credence to the notion that intestinal epithelium-like phenotypic characteristics are retained in the malignant transformation taking place within the teratomatous elements. The identification of these markers would benefit clinicians in diagnostic and treatment planning. In order to correctly classify the histological subtype of adenocarcinomas, immunohistochemical profiling is essential. According to Park *et al* (28), CDX2 expression was found in ~60.9% of gastric adenocarcinoma patients, highlighting the diagnostic value of organ-specific immunohistochemical markers in differentiating between adenocarcinoma origins. The immunohistochemical profile, CDX2-positive, CK7-positive, TTF-1-negative and CK20-negative, produced a positive predictive value of 85.7% for signet-ring cell adenocarcinoma in a documented case of the cancer developing within a teratoma, underscoring the usefulness of immunohistochemical markers in determining the tissue of origin (29).

Koseoglu *et al* (30) demonstrated that adenocarcinoma originating from colon glands in the testis exhibited CEA(+), CA 19-9(+), CK20(+) and CK7(-) based on immunohistochemical staining, which was distinct from the findings of the case presented herein. Asano *et al* (31) reported that in patients with non-metastatic testicular malignant transformation, no recurrence occurred following radical orchiectomy. However, in cases with metastasis, the prognosis of testicular malignant transformation is heavily influenced by the histological phenotype. Testicular malignant transformation with adenocarcinoma typically requires an aggressive treatment regimen. Kasai *et al* (27) described a patient who succumbed to malignant transformation 8 months following orchiectomy despite receiving cisplatin-based chemotherapy. In the case in the present study, metastatic lesions continued to progress despite combination chemotherapy based on cisplatin. These observations highlight the importance of discussing potential confounding factors and limitations in treatment efficacy. Thus, the early detection of malignant transformation is crucial for improving patient outcomes. Moreover, incorporating a dedicated section on study limitations and potential confounders would strengthen the discussion and provide a more balanced interpretation of the findings. Previous research has shown that somatic-type malignancies arising in metastatic GCTs are associated with a significantly worse overall survival compared to those confined to the testis, with carcinomatous subtypes carrying the highest risk for mortality (32). Somatic-type malignancies are observed in ~2.5 to 8% of testicular GCTs, most commonly arising within teratomas and often presenting in metastatic sites, where they are associated with poor clinical outcomes (33,34).

The present study has several limitations that should be acknowledged. First, as a single case report, the findings cannot be generalized to all patients with testicular teratomas undergoing malignant transformation. The rarity of such transformations, particularly into adenocarcinoma, limits the ability to establish standardized treatment protocols or prognostic markers. Second, although immunohistochemical analysis was utilized to identify the histological subtype and probable origin of the malignancy, genomic or molecular profiling was not performed, which could have provided deeper insights into the mechanisms of transformation. Third, the temporal association between chemotherapy and malignant transformation could not be definitively established, leaving causality speculative. Finally, due to the retrospective and descriptive nature of this report, long-term outcomes beyond the presented follow-up period remain unknown. These limitations underscore the need for multicenter data collection and molecular studies to better understand the pathogenesis and clinical behavior of malignant transformations in testicular GCTs.

In conclusion, the malignant transformation of testicular teratomas, particularly into adenocarcinoma, represents an exceptionally rare and diagnostically challenging entity with significant therapeutic implications. While somatic malignancies, such as sarcomas and enteric adenocarcinomas have been reported in mature teratomas, such transformations in testicular teratomas are exceedingly rare. The mechanisms driving these transformations, including differentiation of embryonal carcinoma cells or secondary changes induced by chemotherapy and radiotherapy, remain under investigation. Given the rarity of such cases, further research is warranted to elucidate the molecular and genetic pathways involved in somatic transformation, particularly those leading to adenocarcinomatous differentiation. The presence of somatic malignancies complicates clinical management, requiring detailed histological and immunohistochemical evaluation. The prognosis depends on the histological subtype, with adenocarcinoma variants often necessitating more aggressive treatment. The present case report highlights the practical importance of vigilant long-term follow-up, the integration of histopathological expertise and timely intervention, particularly in patients with recurrent retroperitoneal masses despite normalized tumor markers. Early detection through tumor marker monitoring and imaging is crucial, and a multidisciplinary approach is essential for optimizing treatment outcomes and improving survival rates.

## Data Availability

The data generated in the present study may be requested from the corresponding author.
